# Restoration of Attention by Rest in a Multitasking World: Theory, Methodology, and Empirical Evidence

**DOI:** 10.3389/fpsyg.2022.867978

**Published:** 2022-04-01

**Authors:** Frank Schumann, Michael B. Steinborn, Jens Kürten, Liyu Cao, Barbara Friederike Händel, Lynn Huestegge

**Affiliations:** ^1^Mittweida University of Applied Sciences, Mittweida, Germany; ^2^Department of Psychology, University of Würzburg, Würzburg, Germany; ^3^Zhejiang University, Hangzhou, China

**Keywords:** rest breaks, attention restoration theory, cognitive resources, mental fatigue, ego depletion, multitasking, energy management, motivated cognition

## Abstract

In this work, we evaluate the status of both theory and empirical evidence in the field of experimental rest-break research based on a framework that combines mental-chronometry and psychometric-measurement theory. To this end, we (1) provide a taxonomy of rest breaks according to which empirical studies can be classified (e.g., by differentiating between long, short, and micro-rest breaks based on context and temporal properties). Then, we (2) evaluate the theorizing in both the basic and applied fields of research and explain how popular concepts (e.g., ego depletion model, opportunity cost theory, attention restoration theory, action readiness, etc.) relate to each other in contemporary theoretical debates. Here, we highlight differences between all these models in the light of two symbolic categories, termed the resource-based and satiation-based model, including aspects related to the dynamics and the control (strategic or non-strategic) mechanisms at work. Based on a critical assessment of existing methodological and theoretical approaches, we finally (3) provide a set of guidelines for both theory building and future empirical approaches to the experimental study of rest breaks. We conclude that a psychometrically advanced and theoretically focused research of rest and recovery has the potential to finally provide a sound scientific basis to eventually mitigate the adverse effects of ever increasing task demands on performance and well-being in a multitasking world at work and leisure.

Everyday wisdom tells us that multitasking is great in the kitchen when a cook is preparing several dishes at once, for example, the chicken to be ready at the same time as the rice, but it becomes worse when trying to schedule the work day (Salvucci and Taatgen, 2011, pp. 3–14). In fact, people’s attentional capabilities are increasingly strained by environmental factors such as time pressure or multiple task demands (Levine, 1998), or even professional requirements (Strobach et al., 2015; Häusser and Mojzisch, 2017). Since multitasking demands preoccupy large parts of people’s daily routines, the question of how to manage or to recover from the strain imposed by overload has become increasingly important, both for researchers and practitioners (Kaplan, 1995; Kahneman et al., 1999; Proctor and Capaldi, 2008). However, despite a multitude of published papers, current research cannot provide answers to fundamental questions. Here we dramatize the position that both theory and methodology for studying the restoration of attention by rest is in a lacking condition at present. Formally, rest breaks are defined as temporal interruptions of an activity, serving the purpose of regenerating mental functions. Conceptually, there are three fundamental aspects that are connected to taking a break, depending on the particular context: to find distance, to change activity mode (e.g., from thinking to sensing), and to recover or regain energy levels (Kaplan and Kaplan, 1989; Colzato et al., 2012; Häusser and Mojzisch, 2017).

## 1. Introduction

In everyday language, rest breaks play an important role both at work and in active leisure time (Fritz and Sonnentag, 2006; Wendsche and Lohmann-Haislah, 2017). The metaphorical nature of everyday language already provides clues about an underlying hypothetical “mechanism” that people perceive as such in purely phenomenological terms. For example, people often talk about “*refueling*” their energy or “*recharging*” their batteries, which clearly implies a kind of resource that diminishes under strain and is restored through rest (Hobfoll, 1989; Kaplan, 1995; Fritz et al., 2011; Zacher et al., 2014). On the other hand, people often say *“I’m fed up with it*,” indicating a state of aversion to be reduced by taking some distance from the ongoing task (Lewin, 1928; Demerouti et al., 2001; Mojzisch and Schulz-Hardt, 2007; Kurzban et al., 2013). Therefore, rest-break structures in working life are firmly established by government law and specified by labor legislation. Although rest breaks in private life can be taken rather flexibly, even there a rhythmic structure can be observed, consisting of a change from strenuous activity to rest and vice versa (Tucker et al., 2003; Monk, 2005; Wendsche and Lohmann-Haislah, 2017). Because daily routines are similar for the majority of people, enabling similar experiences, hardly anyone would not agree with the proposal that breaks have a positive effect on feelings or mental performance (Poffenberger, 1928; Bills, 1943; Wyles et al., 2016). However, such an initial consensus would certainly not last long but maybe even turn into a point of contention if the question is taken further of how *exactly* rest affects cognition in a particular situation.

Though there are numerous proposals and theoretical notions in the scientific literature about how mental fatigue occurs, what demands create it, and how breaks regenerate or even restore it afterward (Strack and Deutsch, 2004; Fritz and Sonnentag, 2006; Wells and Matthews, 2015). Although it is obvious that the underlying mechanisms of rest and recovery might be completely different in the variety of contexts and time scales where strain and recovery take place, this aspect is not sufficiently distinguished in the empirical literature. On the other hand, most of the theoretical approaches are relatively similar in their base assumptions while focusing on rather specific contexts or making predictions about quite different units of observation (e.g., objective test performance vs. subjective ratings of feelings or motivation). They can be classified into two basic categories that clearly correspond to common everyday metaphors, which we will term here the “*resource model*” and the “*satiation model.*” The resource model covers all proposals that assume a hypothetical reservoir of energy, either perceived as such by an individual or indicated through performance, which depletes through work, is replenished by rest, and can be conserved to some degree by adopting strategy (Hobfoll, 1989). The satiation model covers those approaches that base their starting point on feelings that include a spectrum of aversive experiences capable of inhibiting ongoing task operations (Lewin, 1928; Watson et al., 1988; Thayer, 1989; Tellegen et al., 1999; Langner et al., 2010; Matthews, 2021).

The goal of this paper is threefold. First, the observable phenomena are to be ordered and classified, followed by a theoretical analysis of pauses and their effects on performance. Finally, empirical studies are discussed and methodological aspects elaborated on how pause effects can be meaningfully investigated by means of reliable performance-based experimental methods (Steinborn et al., 2018). Here we are focusing on experimental rest-break research, while considering field research (employing mostly correlational methods) with respect to similarities and differences in both the theorizing and methodological approaches. Since research on rest and recovery is a relatively broad and interdisciplinary field, relevant to many scientific domains including sports sciences, school psychology, work and occupational psychology, and cognitive-experimental psychology and neuroscience, it is rather impossible to organize the manuscript in the style of a classic meta-analysis, where study results are statistically aggregated to generate a quantitative estimate of an empirical phenomenon (e.g., the rest-break effect, etc.). Specifically, while it is viable to aggregate studies in well-defined work field situations, like the aftereffect of lunch breaks on performance (Monk, 2005), or the effects of shift-work on well-being (Kantermann et al., 2007, 2012), considering variations across studies as random factor, this is neither possible nor feasible in a purely experimental situation. This would actually reduce a rather complex research question to whether an effect exist or not (or what size an effect is on average) while ignoring crucial aspects of theorizing, design, and measurement methodology that is absolutely crucial for a deeper understanding of behavioral phenomena. Therefore, the present work aims at analyzing the problem at the level of theory, methodology, as well as psychometric measurement in the light of existing empirical evidence.

## 2. Taxonomy of Rest-Break Structures

It is virtually impossible to theorize on the effect of rest breaks on mental function without considering the variety of contexts and time scales where breaks are relevant (Tucker et al., 2006; Helton and Russell, 2015; Wendsche and Lohmann-Haislah, 2017). In labor law, break systems are anchored and regulated by legislation. Work breaks are defined as the period of time specified in the company agreement during which employees’ work performance is suspended. Although statutory work breaks are primarily implemented for the purpose of taking meals, they also have a designated recreational function (Lombardi et al., 2014; Paech et al., 2014; Pylkkonen et al., 2015; Roach et al., 2016). During this time, employees are neither required to perform work nor to be ready to do so, and even more, they are free to decide where and how to spend this time. Work breaks can thus be spent both at the workplace and outside. In the conception of labor law, the break is a state of inactivity inserted into a work process, where inactivity also concerns the attitude toward the work performed in each case. Thus, inactivity is limited to the work process itself, so that any activities unrelated thereto, such as reading the newspaper, listening to music, or exercising, constitute a break activity. Scholz et al. (2017) conducted an experimental field study comparing different types of breaks and found that the exact type of break is of less relevance than the sole fact that a break takes place at all (see also Helton and Russell, 2015; Steinborn and Huestegge, 2016; Wendsche and Lohmann-Haislah, 2017).

By nature, rest breaks can be classified according to various aspects and dimensions, such as the time scale or context where rest is taken (see Table 1). An important aspect refers to the distinction between experimental rest-break research that typically takes place in the laboratory (using student-based participants) and the field-research approach that takes place within the facilities of a company (using employees as participants). Experimental studies typically manipulate critical experimental variables (e.g., duration, task, and content, etc.), field studies are often based on correlational methods. As a consequence, equally sounding theoretical concepts (e.g., ego depletion vs. burnout depletion) often differ in their exact meaning and likely address a rather different underlying mechanism, as compared to those addressed in experimental studies. Finally, field studies typically base their conclusions on self-report measures of mood or mental fatigue, obtained via questionnaire, or asking about the frequency of taking short breaks during the work day (Krajewski et al., 2010; Fritz et al., 2011; Zacher et al., 2014; Kim et al., 2017, 2018). In contrast, experimental studies are typically aimed at assessing performance differences (e.g., measuring the speed and accuracy of mental work) as evoked by the manipulation of critical experimental conditions. Some studies use a combined experimental-correlational approach in field settings, studying the effects of rest on workers’ performance using laboratory tasks aimed to simulate the micro-case of the work process, though it is difficult to generalize (or transpose) the effect of rest on performance in a laboratory task on the real work process (Scholz et al., 2017, 2019).

**TABLE 1:** Overview and description of paradigms and phenomena closely related to rest-break research.

	Type	Design and calculation	Assumption and description
**1**	Post-lunch dip	Pre-post comparison of performance before vs. after the main lunch break	- performance decline after lunch - glucose intake ≥ insulin response ≥ fatigue - digestion impose (“*dual-task*”) interference - likely a part of diurnal rhythms
**2**	Time-of-day effect	Differential daytime performance curves as a function of work-break schedule	- evidence for both diurnal trends and fluctuation - empirical evidence is mixed and contradictory - lack of proper design in the majority of studies - difficulties of implementing proper controls - confounds: test-taker effects and practice
**3**	Incubation effect	Comparison of problem solving performance after resting vs. no-resting vs. interference	- improved problem solving after resting - latent processing during rest ≥ restructuring - the function of rest is to reduce fixation - can be conceived of as a rest-break paradigm
**4**	Memory consolidation	Comparison of memory recall after resting vs. interference	- relatively mixed empirical evidence - is an interference paradigm in a strict sense - rest = control, a proxy for “non-interference” - not a rest-break paradigm in the proper meaning
**5**	Restart-cost effect	Costs of re-starting mental set as a function of lengthening rest breaks (or unexpected task onset after long rest)	- concerns the detrimental effects of long intervals - benefits of rest turn into costs when too long - theoretical objective: aspects related to forgetting - has a more specific meaning in task-switch literature
**6**	Interruptions	Comparison of memory recall after interrupted vs. non-interrupted tasks	- evidence for increased recall of interrupted tasks - prerequisite: completable, purposeful tasks - use of intrinsically motivating task forms - examples: Hungarian cube, puzzles, etc.
**7**	Delays	Comparison of performance in a no-delay vs. predictable delay vs. non-predictable delay condition	- concerns “unwanted” delays during workflow - example: computer loading bar; CPU overload - aimed at simulating workflow interruptions - contextual semantics differ from rest-break studies

*The types 1–2 are paradigmatic approaches to study daytime change and its compensation by rest in the context of work and leisure; the types 3–4 are not concerned with mechanisms of recovery but with latent processes of consolidation and representational restructuring. The types 5–7 address specific functions of inserted time intervals (e.g., forgetting as task set, motivation to complete a puzzle, annoying effects of computer loading bars or CPU overload, etc.).*

Crucially, rest breaks must conceptually be distinguished from other types of interruption periods. For example, preparatory activities or waiting times in performance tests are typically not considered rest breaks, even when they are not overtly performed, such as monitoring or other kinds of watchkeeping activities (Warm and Alluisi, 1971; Steinborn and Langner, 2012; Ross et al., 2014). For example, Broadbent (1971) argued that rest break time is to be distinguished from preparatory time, though it might depend on the particular context whether individuals actually recruit rest intervals for preparation (Rabbitt and Vyas, 1980; Steinborn and Langner, 2012; Langner et al., 2018). More generally, all kinds of active waiting periods that require vigilance and where complete goal detachment is not possible are not to be considered resting time (Langner and Eickhoff, 2013; Thomson et al., 2016). In a practical sense, work breaks can be divided into work interruptions of different length as well as of different functions; from a cognitive-psychological point of view, however, the most meaningful is the division into three approximate time categories (or time zones, respectively): The long break (30–60 min) represents the break for meals and is the most relevant break in work contexts. The short break (3–10 min) is actually a form of break where the recreational aspect is paramount, and the majority of studies are actually addressing this kind of rest break. The last category concerns micro-breaks (<3 min), which are extremely brief pauses that mainly serve to reduce short-term overload of the cognitive system. While the study of long breaks is restricted to field approaches, the latter types are typically examined via experimental-design (Adams, 1954; Rickard et al., 2008; Ariga and Lieras, 2011; Helton and Russell, 2015; Ralph et al., 2016; Steinborn and Huestegge, 2016).

As already indicated, the *long break* is the most relevant break in the regular working life. It is an integral part of the classic 8-h workday and can only be investigated in this context (Chmiel et al., 1995; Folkard, 1997; Monk, 2005). It is neither possible nor feasible to manipulate critical experimental conditions in real-work contexts, such as to vary the break’s length and content, so only the observation of those aspects that are naturally occurring during the workday remain suitable for investigation (Lombardi et al., 2014). Thus, the full-scale study of performance patterns over the entire workday is a classic domain of field research, often combined with a correlational approach (Meehl, 1967; Fritz and Sonnentag, 2005). Accordingly, the results of studies on time-of-day effects are also difficult to interpret, as numerous methodological artifacts can hardly be avoided (Roach et al., 2016; Riley et al., 2017). There are two relatively well-established effects that seem paradoxical at first glance but can be explained quite easily on closer inspection, (1) the time-of-day effect and the (2) post-lunch dip phenomenon. The *time-of-day effect* describes, according to mostly earlier studies, an increase in performance over the course of the day, at least better performance in the afternoon relative to morning times (Folkard, 1975; Roenneberg et al., 2003). More recent studies acknowledge the difficulty in studying daytime trajectories as there are many confounding variables that cannot be controlled easily. For example, because it is difficult to avoid the use of a repeated-measures design, daytime trajectory effects of performance are often superimposed by artifacts such as test-taker effects or practice gains (Folkard, 1975; Ballard, 1996; Dinges et al., 1997; Flehmig et al., 2007b; Lim and Dinges, 2008; Langner and Eickhoff, 2013; Basner et al., 2018; Steinborn et al., 2018).

In some way, the empirical finding of a *post-lunch dip* phenomenon contradicts the predictions implied by a resource–recovery model, as it refers to a decline (not an improvement) in performance immediately after (meal) breaks. It is also at odds with the predictions of prominent models connecting small declines in glucose levels during a task with decreased willpower and mental performance (Gailliot et al., 2007; Vadillo et al., 2016). To study lunchtime effects, one or more critical groups are typically compared with a control group each before and after the experimental variation (i.e., the break including the meal). In general, performance costs are found in the critical relative to the control group, and this effect is influenced by numerous factors. High calorie diet or high carbohydrate diet are the most important determinants of the effect, and even though there is a great heterogeneity with respect to the particular tasks and performance measures, empirical findings seem relatively robust (Bes et al., 2009; Reyner et al., 2012; Debus et al., 2014). Nevertheless, a detailed comparison of results across studies remains difficult because of the large differences in the use of tasks and performance metrics, as most of them hardly meet current psychometric standards (cf. Langner et al., 2010; Miller and Ulrich, 2013; Steinborn et al., 2018). Monk (2005) argues that there could be a habitual component in the post-lunch dip effect, since even without food intake there is often a small performance drop in the early afternoon, similar to the post-lunch phenomenon. However, the empirical evidence is rather unclear, as only a few studies have included such a condition.

The *short rest break* (3–10 min) and its effects on performance is typically studied via the experimental approach, typically with a clear focus on the underlying cognitive processes. Roughly speaking, the research approaches can be divided into three categories, corresponding to which three basic types of tasks are used. (1) Active sustained attention is mostly measured by means of speeded tests, sometimes termed continuous–performance tests, or mental–concentration tests (Krumm et al., 2008; Blotenberg and Schmidt-Atzert, 2019a,b). These tests require continuous processing and are highly demanding at a subjective level (Pieters, 1983, 1985; Van Breukelen et al., 1995; Mojzisch and Schulz-Hardt, 2007; Steinborn et al., 2018). Notably, this type of task is also relatively often used to experimentally induce a hypothetical mental state termed *ego depletion*, which is relatively popular among social psychologists (cf. Hagger et al., 2010; Vohs et al., 2021). Speeded self-paced tests typically exhibit the highest degree of test reliability, thus a high number of items can be administered per unit of time, enabling precise measurement (Van Breukelen et al., 1995; Steinborn et al., 2018). (2) A rather passive type of sustained attention (vigilance) is measured in a classical way with detection tasks, which require the individuals to keep track on watching for rarely presented targets, either in time or among distractors (Mackworth, 1948; Warm et al., 1974; Langner et al., 2011), which is less reliable because target items are spaced by an intertrial interval thus only a few (1–2) responses are registered per unit of time. More recent studies opted for the use of the psychomotor vigilance test (PVT), which requires speeded responses to a simple targets, spaced by long and variable waiting intervals. Research indicate that these interval are not perceived as a rest but to form expectations about temporal moments to which the participants are to give a speeded response (Wilkinson, 1959; Langner et al., 2010; Steinborn et al., 2016; Massar et al., 2018; Unsworth and Robison, 2019).

The basic design for studying short rest-break effects consists of the following components (cf. Steinborn and Huestegge, 2016): A baseline condition is used to propagate mental fatigue (e.g., a test of 20–40 min), which is compared to one or more experimental conditions where rest-breaks (of 1–5 min) are intercalated. Typically, not merely the group differences (Ariga and Lieras, 2011; Helton and Russell, 2015) but a differential time course in the rest condition as compared to the baseline condition is taken as indication that rest prevented fatigue from accumulating (see Table 3). It is important to note that the term “mental fatigue” is most often not used in a specific sense but rather referred to as an umbrella term (cf. Langner et al., 2010), and the test length to propagate fatigue should not exceed 60 min. This might seem counterintuitive at first glance, however, many early studies have administrated their individuals to perform tasks (mostly mental arithmetic) over longer periods of 4–8 h, showing a decline in performance after 30–60 min, which then leveled off at a low performance (e.g., Robinson and Bills, 1926; Manzer, 1927; Schubert, 1932; Bills, 1943). Typical research questions involve a comparison of different types of rest, for example, whether the rest is taken in an active (i.e., walking) or passive way (sitting) or in the same vs. changing environments, or with respect to the freedom of choosing how to spent the given time for rest (e.g., Ulrich et al., 1991; Korpela and Hartig, 1996; Ross et al., 2014; Steinborn and Huestegge, 2016; Basu et al., 2018; Pasanen et al., 2018; for theoretical considerations).

The *micro-break* effect refers to the phenomenon that even the shortest pause inserted during continuous cognitive activity typically yields significant performance gains, relative to a condition where no such pauses are given (Adams, 1954, 1955; Eysenck, 1965, 1969). Studies theorizing on micro-break effects can be divided into at least two categories with assumedly distinct underlying mechanisms. Studies on the so-called “reminiscence” effect typically use continuous–performance tests, and there are also studies where pauses are not conceptualized in terms of restoring energy but as brief intermission phases that serve coordinating mental structure during memorization (Bower and Springston, 1970). While the former is typically concerned with (motor-)learning effects (Adams, 1954; Rickard et al., 2008), studying performance gains as a function of rest, the latter is concerned with the accumulation of short-term fatigue, sometimes termed accumulated refractoriness, and its reduction through rest (Weaver, 1942; Bertelson and Joffe, 1963; Rasch, 1980). In a typical study, tasks were presented as a continuous series as is common in psychometric instruments of the speed-test type (Rasch, 1980; Steinborn et al., 2018). In such a situation, one can observe occasional “mental blockades” occurring during continuous task processing. Bills (1931, 1935) studied this phenomenon in numerous task forms such as addition, coding, or sorting, which are the most common classes of items in speed tests (Neubauer and Knorr, 1998; Flehmig et al., 2007a; Wühr and Ansorge, 2019). His analyses of individual responses showed that even after 5 min of uninterrupted work, mental blocks could be observed, which were defined as extremely slow reactions relative to the average. According to Bills, mental blockings can be conceived of as enforced (or system-generated) pauses, aimed for refractoriness to dissipate. If one would administrate smallest breaks preventively, by inserting distributed brief pauses, then the blocking phenomenon is typically reduced or absent (cf. Van Breukelen et al., 1995).

## 3. Related Phenomena

### 3.1 Incubation

As mentioned earlier, rest breaks are distinguished from other forms of intervals separating work periods in time (e.g., preparation, monitoring, etc.), and other type of activities that require the further maintenance of attention (Gillie and Broadbent, 1989; Goschke and Kuhl, 1993; Allport and Wylie, 2000). A characteristic of these aspects is that they be distinguished theoretically while the empirical test of prediction derived from theory depends on the quality of design and measurement precision. However, there are some related phenomena obtained in a paradigm similar to a rest-break situation (at least could be framed as such) but with an entirely different underlying mechanism. One example is the so-called incubation paradigm, where individuals are typically administered with a problem-solving task, with the solution being dependent on sudden insight that is often prevented due to fixation or when misdirected toward another pathway (e.g., Vul and Pashler, 2007; Bilalić et al., 2008; Sio and Ormerod, 2009). The incubation effect is demonstrated by comparing a condition where individuals are administered to take a rest, relative to an alternative (distracting) activity and a control (no-rest) condition. The outcome, relatively often shown, is that those participants (a) who took a rest during an incubation period performed better than (b) those who performed a task during this period, (c) relative to the baseline (no-rest) condition, though opposing findings have also been reported (Sio and Ormerod, 2009).

### 3.2 Episodic–Trace Consolidation

Another related line of research concerns studies on memory consolidation during nocturnal sleep, daytime napping, or other kinds of resting periods. The term memory consolidation refers to a category of processes considered to support the stabilizing of a memory trace after its initial acquisition. Importantly, psychological research is not so much concerned with low-level consolidation processes such as synaptic consolidation, which has clearly been shown to occur 1–2 h after initial learning. Instead, the focus is on cognitive consolidation, that is, on how mental representation are formed or restructured, and how episodic traces are finally transformed into abstract codes with flexible retrieval structures (Tulving and Thomson, 1973; Hintzman, 1974, 1986; Tulving and Watkins, 1975). Classically, memory consolidation is studied during sleep, though psychological research is more focused on shorter time scales, studying mnemonic consolidation after resting relative to an active-work condition (Wamsley, 2019; Martini and Sachse, 2020). The participants in such studies are typically required to memorize material, with recall performance being tested afterward at a later time point, under a relaxed condition (e.g., closed eyes, napping, etc.), relative to a distractor condition (e.g., mental work, watching videos, etc.). The theoretical prediction is that waking rest supports the consolidation of previously learned memory content, relative to an interference condition, though the empirical findings are rather mixed with this regard (Martini et al., 2019). While this line of research addresses the aspect of “resting” on memory consolidation, it is important to understand that the *de facto* research question in this field is not on how rest breaks restore attention, but more on the *benefit of non-interference (vs.* interference) on memory recall performance (Wamsley, 2019; Martini and Sachse, 2020).

### 3.3 Restart Costs

By definition, the term restart costs refer to a cost point that is incurred when the time taken for rest is too long, and in this way, it could be viewed as a non-beneficial effect of rest on performance. It is directly connected to the question of how long a rest break should be to achieve the best results, which has been debated already by earlier studies (Graf, 1922; Manzer, 1927; Poffenberger, 1928; Schubert, 1932; Barmack, 1939; Ross and Bricker, 1951). In the literature on rest-break research, this is studied by experimentally varying the rest-break length and to determine the functional relationship between rest length and recovery. In the specific literature on task-switching performance, the term restart costs is not in the same way conceived of as a rest break, but taken in the more restrictive sense as the relative costs of intercalated time intervals in task repetition relative to alternation trials (Allport and Wylie, 2000; Wylie and Allport, 2000). In this way, re-start costs in task-switching research are linked to aspects of forgetting, or other aspects of losing proper memories for the upcoming task (Altmann, 2002; Altmann and Gray, 2008; Kiesel et al., 2010; Vandierendonck et al., 2010; Vallesi et al., 2013). We therefore argue that the conception of re-start costs are in its specific form (as used in the task-switching literature) different from those studied in the rest-break literature. While the rest-break literature deals with the aspect of attentional replenishment, the task-switching literature is concerned with aspects of forgetting, and in this way, re-start costs are not the primary concern but a side-show phenomenon in the task-switching literature.

### 3.4 Interruptions

The concept of rest breaks exhibits some remarkable similarity with the concept of an interruption, which refers to a temporal interruption of an activity that is not necessarily needed, and neither wished, nor intended, and not expected as such at a certain time point during ongoing task performance. In the early literature that has its starting point in Lewin’s (1928) field theory, it was shown that when individuals were interrupted during an ongoing task (stringing beads, solving puzzles, etc.), but were allowed to continue with other tasks, then the interrupted task was recalled more often than the uninterrupted ones (Zeigarnik, 1927). Even more so, if they were allowed to freely decide what do to at some point, the individuals tended to re-start and completing the interrupted tasks (Ovsiankina, 1928), which indicates that the memory for non-completed tasks tend to further persist in memory and thus guiding ongoing decisions (Gillie and Broadbent, 1989; Goschke and Kuhl, 1993; Einstein and McDaniel, 2005). Thus, the study of (completable) task interruptions like solving a puzzle is an interesting counterpoint to the study of rest breaks because it demonstrates the energizing effect of intrinsic motivation on cognitive persistence such that individuals are prevented to perceive a need for rest before completing a task such as a puzzle, or when they are given an objective (or purpose) to aim for Kruglanski et al. (2012), Suri et al. (2015), Krishna and Strack (2017), Steinborn et al. (2017).

### 3.5 Delays

While the theorizing in the rest–recovery model literature is focused on mechanisms of attentional replenishment, considering rest as to result in an improvement or at least stabilization of performance, the literature on delayed processing or unpredicted interruptions during ongoing action sometimes shows the opposite effect, as often a detrimental effect on performance is reported which is considered to originate from kinds of distraction. In fact, delays can severely interrupt workflow and may also result in affective responses, or emotional disturbances, such as increased distress, despite a measurable decrease in performance (Kohlisch and Kuhmann, 1997; Szameitat et al., 2009; Thomaschke and Haering, 2014). A crucial difference between the research on rest breaks and those on interruptions lies in the contextual semantics of situational prototypes where breaks or interruptions typically take place. By definition, rest breaks are studied in situation where rest is naturally indicated such as to counteract the time-on-task effect in sustained-attention and vigilance tasks. In contrast, interruptions are meant to disrupt the workflow and are typically infused in situations where they are unwanted. While people can clearly distinguish between both in everyday situations, it is difficult to determine the contextual semantics of everyday concepts in the artificial situation of a laboratory experiment. For example, the loading bar in computer games would unlikely be conceived of as a rest break, but when one attempts to study this situation in the laboratory experiment, it becomes difficult to distinguish (Suchotzki et al., 2017; Suchotzki and Gamer, 2018).

## 4. Theories of the Rest-Break Effect

The starting scenario of a theory of pause effects consists of the observation that individuals show a decline in performance during the processing of continuous task forms already after a relatively short period of time (Nuechterlein et al., 1983; Caggiano and Parasuraman, 2004; Langner et al., 2010; Steinborn et al., 2016). Depending on the type of task form, this decline manifests itself in slower reactions, higher error rates, or stronger work fluctuations, but in any case in a reduced efficiency of work performance (Bills, 1935; Barmack, 1939; Craik, 1948; Rohmert, 1973a,b). By inserting a break condition, it is possible to test the extent to which this drop in performance is diminished or reversed (Steinborn and Huestegge, 2016). The so-called tester fatigue effect does not just occur after hours of prolonged mental work, but rather emerges quickly; according to Bills (1943) the first signs are already recognizable after 5 min. In aggregated performance characteristics, clear-cut effects are determined after 10–20 min (Bills, 1931, 1935). It reported from numerous studies that individuals in pure (non-speeded) detection tasks exhibit the primary performance drop in the detection rate within the first 20 min, which then levels off asymptotically at some point (Frankmann and Adams, 1962; Langner et al., 2011; Langner and Eickhoff, 2013; Thomson et al., 2016). When reaction time-based task forms are used, a similar pattern emerges in the form of slowed reaction times, but here the drop in performance is often superimposed (to varying degrees depending on the task form) by practice. Based on a study by Thomson et al. (2014), this aspect can be well explained: comparing the performance trajectories of two task forms (cf. Figures 1, 4, complex vs. easy task form), a seemingly “paradoxical” performance gain in the former but expected performance costs in the latter task are revealed. Yet, the self-reports clearly show an increased tendency toward mind wandering in both tasks, a typical indicator of decreasing attentional control (Smallwood and Schooler, 2006; Smallwood, 2013; Thomson et al., 2015, 2016).

Before theorizing on the potential mechanisms underlying the rest-break effect, the main types of models in the domain of sustained attention must be characterized and distinguished (see Table 2). They may be categorized along three dimensions, (1) the assumption of a volume of cognitive resources vs. a degree of experienced mental satiation, (2) the corresponding dynamics with which these resources are utilized, exhausted, and replenished, (3) and the extent of strategic control over the deployment of resources over time. In this context, it is difficult to assign taxonomically rigorous categories to the theoretical approaches published in the literature because they are sometimes not clearly articulated, contain inconsistencies or even contradictions in their propositional systems, or lack a specified measurement model (cf. Kahneman and Miller, 1986; Rothermund and Wentura, 2010; Greenwald, 2012; Stroebe and Strack, 2014; for a discussion). In particular, authors of empirical papers often argue in a way that consists of a diverse mixture of model predictions, everyday metaphors, and platitudes. The classic *resource-volume model* could be considered a standard model because it is straightforward, enables clear prediction, and is well suited as a starting position. The resource-volume model has its roots in Kahneman’s energetic capacity model (Kahneman, 1973). While less concerned with how resources can be shared across tasks (Meyer and Kieras, 1997a,b; Tombu and Jolicoeur, 2003; Wickens, 2008), it focused more on the distribution of resources over time, which renders it particularly interesting from a rest-break perspective. Regarding the underlying dynamics, the basic assumption is that ongoing activity leads to a decrease in volume of resources, while pauses lead to an increase in volume (see following section for more details). The *satiation model*, in contrast, uses an entirely opposite metaphor. Here, continuous activity causes an increase in mental satiation, while taking breaks reduces the same (Mojzisch and Schulz-Hardt, 2007). The dynamics or momentum by which these parameters are drained and replenished is also an important parameter. Most current approaches implicitly assume a constant and slow decrease in resources over time, often (automatically) inferred from averaged performance curves in experimental conditions.

**TABLE 2:** Popular metaphors typically guiding theoretical predictions in the rest-break literature.

	Type	Metaphor and symbolic assumptions
**1**	Energetic-resource model	A hypothetical reservoir of resources is depleted through mental work (e.g., with time on task) and replenished during rest. The state of resource disposal is indicated by the decrease in performance speed in the task over time.
**2**	Strategic-resource model	Though resources are depleted during an ongoing sustained-attention task, they can be held in reserve or can be distributed in flexible ways. Thus, a straightforward relation of resource volume and performance over time is no longer assumed. Note: variants of strategic-resource models need further specification in order to be verifiable.
**3**	Ego depletion	Acts of self-control deplete resources and might potentially be replenished through periods of rest. The typical experimental arrangement goes as follows: Resources are depleted in task A and tested in a subsequent task B. In this way, ego depletion is concerned with the sequential transferability of a depleted across two subsequent tasks.
**4**	Satiation model	The critical variable relevant to performance is not a hypothetical volume of resources but the level of accumulated satiation that is experienced as aversive, thus considered the main driving force of behavior. Perceived satiation increases during repetitive work and dissipates during rest.
**5**	Reactive inhibition (Rasch model for speed tests)	Processing repetitive tasks yield a resistance gradient against further continuing with the ongoing action, conceived of as a distraction tendency. The inhibition gradient increases with prolonged task processing and leads to distraction (enforced rest) when reaching a critical threshold. This inhibition tendency thus increases monotonically during task processing and decreases during periods of (a) distraction or during (b) rest breaks.
**6**	Opportunity Costs	The term opportunity costs refers to the potential loss of a missed opportunity as a result of choosing one opportunity and foregoing another. These costs are indicated by the subjective experience of effort or aversion when proceeding with the ongoing task, but are relieved when the task is changed (that is, when alternatives are considered).
**7**	Attention Restoration Theory (ART)	Resources are claimed during the working hours of a day and replenished in the remaining free time and on weekends. Crucial is that recovery is not merely a function of time but depends on the context where rest takes place. Spending time in nature is assumed to be more beneficial than spending time in urban environments. In a strict way, ART is a psycho-sociological model but often misconstrued in the empirical literature.
**8**	Conservation of Resources Theory (CRT)	This is a psycho-hygienic model of stress prevention which is popular in the applied fields of rest-break research. In brief, the theory deals with how people perceive and estimate own resources including the costs of handling anticipated threats and challenges imposed by impending future events, and how people deal with uncertainty, respectively.

*The models 1–3 employ a metaphor (resource volume) with a preconditioning parameter while the models 4–6 (satiation) make use of a delimiting parameter (thus both symbolic classes utilize a diametrically opposing metaphor to each other), though both metaphors make similar predictions. The models 7–8 are, in a strict sense, not performance models but theories about human wellbeing in the context of strain and recovery, though frequently referred to also by the experimental literature on mental fatigue and its recovery by rest breaks.*

**TABLE 3:** Computation and meaning of the four essential contrasts in experimental rest-break designs.

	Type	Calculation and interpretation
**1**	Simple block comparison (relative block difference)	A baseline (no-rest) condition (A) serves to estimate the performance decrement over the testing period without rest breaks. A rests-break (B) condition serves to estimate the performance trajectory when rest is provided. Directly contrasting both yields a measure of the relative A–B block difference in performance, which provides a primitive measure of the overall benefit provided by rest, relative to a continuous condition.
**2**	Global rest-break effect (relative time change)	To obtain a measure of the “relative” change in performance over the testing period, the trajectory of performance (time-on-task gradient) for both A and B is contrasted. This gives an estimate of the relative change in the time-on-task effect in performance. In other words, it informs how the performance decrement is prevented by rest breaks, relative to when no rest break is given.
**3**	Local rest-break effect (before–after rest)	The local effect of rest on subsequent performance is obtained by contrasting the adjacent sections before and after the rest break (pre–post rest comparison). This gives an average estimate of the local benefit of rest that immediately occurs in the time series closely before and after taking a rest, irrespective of the time trajectory.
**4**	Differential effectiveness (early vs. late rest breaks)	To test the assumption that the effectiveness of a rest itself increases with testing time (i.e., with time on task), the local (pre–post) rest effect at different positions during the testing period is directly contrasted. A larger relative effect at late positions in empirical data would indicate that the immediate effect of rest increases over the testing period, in other words, that rest is more effective at late relative to early positions.

*The type 1 is, in a strict sense, not interpretable (see Steinborn and Huestegge, 2016) but frequently used in the literature, thus presented here for reasons of completeness. Type 2 tells how rest compensates a potential performance decrement (the time-on-task effect). Type 3 concerns the local dynamics of recovery and thus provides a measure of how immediate recovery occurs directly after the break. Type 4 gives an indication of a change in the local effectiveness change of (early vs. late) rest breaks.*

The third dimension concerns the degree of strategic control over the available resources (Van der Molen, 1996; Pashler, 1998; Sanders, 1998). The ideal norm of a *strategic-resource model* assumes a more or less flexible resource allocation over time. The perceived experiences of effort are considered the internal indicators that determine the momentary strategy, such that a feeling of “ease” indicates available energy while aversion indicates the need for rest and recuperation (Thayer, 1989; Matthews et al., 2002; Langner et al., 2010). Individuals seem to be able to anticipate effort in advance (in terms of an energetic cost point), which is often referred to as economic-strategic model (Humphreys and Revelle, 1984; Langner et al., 2010; Krishna and Strack, 2017). In contrast, a *non-strategic model* would be based on the assumption that individuals always work at maximum performance or at the individual performance peak. The presumption that individuals work at maximum effort is also an absolute prerequisite in the determination of test performance criteria in classical test theory (Miller and Ulrich, 2013; Steinborn et al., 2018). This is also tacitly presupposed in studies aimed to induce ego depletion, a concept referring to a hypothetical state of complete exhaustion of the resource volume or self-regulatory system. It is assumed that the processing of certain tasks over a defined period of time leads to a partial or complete reduction of the resource volume, that this can be measured by means of certain indicators, and that breaks lead to a replenishment of the resource volume. In this chapter, we evaluate theoretical-model approaches in the light of empirical evidence, but we must emphasize that these ostensibly “competing” model approaches should not be conceived of as alternative explanations, but rather as different cultures (Greenwald, 2012; Stroebe and Strack, 2014) in the empirical approach to the study of pause effects.

### 4.1 Energetic Capacity Model

The energetic capacity concept is one of the central elements in the research field of sustained attention and vigilance (Jennings and van der Molen, 2005; Langner and Eickhoff, 2013). The critical variable is the volume, which is decreased by cognitive work and recharged by breaks. In contrast to the computational concept of resources in dual-task research, the central issue here is not the allocation of a flexible resource to multiple forms of concurrent tasks, nor is it about the allocation of separate pools of resources to tasks with a need for a specific type of resources. Rather, the central issue revolves around the distribution of the capacity that can be provided by the resource over the time during which mental work is performed. In this respect, the energetic resource model rather corresponds to the metaphor of everyday language use (Greenwald et al., 1986; Kahneman, 2013, ch. 2): during intense mental work, the reservoir of mental resources is reduced or emptied, and it is recharged during breaks. The empirical indicator for the state of resource disposal is the decrease in performance in the task over time, measured with the respective characteristic values. The metaphor of an energy reservoir that is discharged and recharged by work or breaks is the core element of almost all theoretical approaches in this area. Differences often relate to the dynamics of these processes, the nature of the effect variables, and the weighting of additional variables such as self-reported motivation or psychophysiological parameters (Langner et al., 2010; Lim and Kwok, 2016; Steinborn and Huestegge, 2016).

Kaplan’s (1995) attention-restoration theory is a highly regarded theoretical approach that (in a close definition) relates to long-term recovery. In a strict sense, it is a psycho-sociological theory that addresses the dynamics of recovery and stress in the context of the real world of work and leisure. An outstanding feature of this theory, relative to other accounts theorizing on rest and recovery, is that it holds clear implications for urban planning (Berman et al., 2008; Atchley et al., 2012; Anguluri and Narayanan, 2017; Pasanen et al., 2018). At its core, the model states that energetic resources are claimed by goal-directed work and restored in free time (during non-goal-directed activity). Yet, recovery is not only a function of spare time, as the model makes a distinction regarding the context where rest takes place. In other words, spending time in nature is assumed to be more beneficial than spending time in (typical) urban environments (but see Ouellette et al., 2005). According to this idea, natural environments are rich in what they term “gentle fascinations,” that is, sensations that can be processed with effortless attention that is automatically directed by stimuli and not tied to goals (Kaplan, 1995; Berman et al., 2008; Kaplan and Berman, 2010). These include, for example, clouds moving across the sky, leaves rustling in a breeze, or water flowing over rocks in a stream. Critical to this approach is the theoretical distinction between reflective and automatic attentional control (cf. Strack and Deutsch, 2004; Krishna and Strack, 2017), albeit related to a more sociologically relevant context. There is also empirical support for his proposal showing that population satisfaction is higher in urbanized areas with green walkable parks than in areas without them, even when relevant variables are statistically controlled (cf. von Lindern et al., 2017, for an overview).

In the perspective of their analytical framework, Kaplan and Kaplan (1989) have identified four key principles of recuperation that can be generalized to the field of break research in general, (1) change of scenery, (2) conceptual distance, (3) fascination, (4) and extent of attracting involuntary engagement. Additionally, one more principle can – but rather indirectly – be extracted, (5) the slowing of pace (Levine, 1998; Hoffmann et al., 2021). In terms of break effects, it can be said that people recover well when they make a change from the work task to the break activity, when it offers sufficient distance from the work activity, when the break activity is beautiful or fascinating, and when it engages attention in a non-goal directed way. Finally, it is especially restful when time pressure is removed. Because these principles have high face validity, they have very often served as the basis for predicting rest-break effects in experimental studies. Despite this inspirational power, some methodological problems arise here. For example, it is unclear to what extent predictions can be transferred from large to small time domains. Related to this, many of the operationalizations chosen in “experimental” settings are questionable because of their reductionistic approach. The fundamental question here is whether reaction-time based performance is appropriate to test these principles (Miller and Ulrich, 2013). Therefore, the inspirational power of the theory consists mainly of its high generality, intuitive plausibility, and the logical consistency of its basic arguments (cf. Greenwald et al., 1986; Greenwald, 2012; Gray, 2017).

The recreational function of temporary slowing of behavior was already recognized by early authors, and is of high relevance in the current theorizing. For example, Bills (1943) already made the argument that during continuous speeded activity, the cognitive system can only operate at a maximum level of performance for a very limited time, until it experiences a transient depletion of the required resource pool, which then manifests itself in mental blocking. Bills (1943) argued that this phenomenon could be conceived of a type of pause enforced by the cognitive system, similar to the pause taken on a scheduled basis (cf. Jersild, 1926; Bertelson and Joffe, 1963). The finding that the frequency of exceptionally long responses increases after a prolonged period of uninterrupted processing (while there are typically no changes in the fastest responses) has often been used as evidence for this cognitive-energetic view (Sanders, 1998, ch. 9). Bertelson and Joffe (1963) generalized this principle of alternation of strain and recovery in continuous activities, arguing that performance in self-paced continuous tasks is heavily affected by the ability of an individual to regulate speed and accuracy for the purposeful completion of a task in such a way that it can be performed with optimal efficiency (Neubauer and Knorr, 1998; Stahl and Rammsayer, 2007; Steinborn et al., 2018). Following this view, numerous authors conceptualized “energetic regulation” a basic ability to attain and maintain a state of general optimal activation for upcoming demands, to set an optimal rhythm, and to maintain this rhythm over the duration of the demand.

### 4.2 Strategic Resource Model

One important aspect of research on rest concerns the rate of recovery from mental activity that takes place during breaks. Connected with this point, it is crucial to determine whether recovery potentially occurs (in a non-registered manner) during the active-task period itself. The early observation by Bills (1931) that periods of “enforced” rest take place during the task itself (and against the instruction to perform best) gives a clear indication that the answer is likely “yes.” However, such an observation challenges the logic underlying a straightforward version of an energetic–capacity model, because differences between tasks or individual differences could be interpreted as differences in the employment of strategies (Pashler, 1998; Sanders, 1998). The question that follows is to what degree individuals have strategic control over the available resources to perform a task (Inzlicht et al., 2014, 2018). For example, individuals seem to be able to either delay (Kunde et al., 2004; Jentzsch and Leuthold, 2006) or to speed-up responses (Strayer and Kramer, 1994; Kleinsorge, 2001; Steinborn et al., 2017) when prompted to do so by proper cues, indicating that there is some flexibility in changing strategy. The strategic-resource idea emphasizes the allocation policy of resources, which refers to the basic principles underlying the distribution of capacity over the activity period. In other words, if one knows that there is a long way to go, one more likely allocates resources in a different way than if the way is expectedly short. Yet, it is difficult to exactly determine the strategies used in a task because there are not only differences between experimental conditions but also differences between individuals and, even more so, individuals might change their strategy over the task in a rather qualitative way, which complicates a straightforward interpretation of performance effects in the light of theory (Vandierendonck, 2017).

In fact, there is psychophysiological evidence for such a demand-adaptive mobilization of (physiological) capacity. A typical finding is that the courses of cardiovascular parameters are completely different in sprinters and long-distance runners, with the former showing an increase in heart rate from about 90 to 120 beats per minute immediately before the start of the race, while the latter showed no or only minor changes (e.g., Faulkner, 1964; Hilton, 1975; Inui, 1987; Baden et al., 2005). This is a remarkable finding in that this energetic mobilization occurs before the actual demand, implying anticipatory behavioral adaptation (cf. Requin et al., 1991; Jennings and van der Molen, 2005). It should be noted that the term “strategic” in this context does not strictly imply that the allocation policy results from reflective planning. Rather, it can also be “triggered” by experiences of effort (cf. Goschke and Kuhl, 1993; Koriat and Goldsmith, 1996; Strack and Deutsch, 2004; Mojzisch and Schulz-Hardt, 2007). More critical is the aspect of prevention, which is in contrast to a straightforward formulation of a resource model presupposing a functional relationship between performance and test length (i.e., the time-on-task effect). Many early authors stated that individuals may not always be completely focused, but that moments of rest occur in between, and that these moments of rest serve a regulatory function. Bills (1931, p. 244), for example, has contended that “*the rest afforded by these mental blocks maintains the objective performance of the individual at an average level.”* Therefore, one might wonder whether explicitly administered (micro-)breaks would reduce block frequency, and whether a specific distribution of such microscopic breaks might lead to an optimization of performance and performance stability (Leth-Steensen et al., 2000; Ballard, 2001; Flehmig et al., 2007a; Langner et al., 2010; Steinborn et al., 2010; Unsworth and McMillan, 2014).

Bertelson and Joffe (1963) gave their participants a continuous four-choice task that lasted for about 30 min and required them to press one of four keys assigned to one of four digits (1–4). Their result also suggests that mental blocks enforce a rest period to ensure efficient performance afterward. While mental blocks were always preceded by a slowdown in response and a deterioration in accuracy, both were followed by a sudden improvement. Unsworth and Robison (2019) examined exactly these unintentional pause structures in the psychomotor vigilance test (PVT). The task requires simple responses to targets which are separated from each other by a random interval separating trials from each other (Langner et al., 2010, 2011; Steinborn et al., 2016; Massar et al., 2018). Even here, where targets are separated in time, a similar pattern to that observed by Bertelson and Joffe (1963) emerged, likely because the individuals are engaged in monitoring the time flow until target occurrence (Miller and Schröter, 2002; Steinborn and Langner, 2011, 2012). Lapsing occurred more or less in a rather periodical way and was not related to the time position as given by the length of intervals. In some way, this confirms Broadbent’s assumption that preparatory time (and any watchkeeping time in vigilance tasks) is not resting time but a rather arduous mental state of effortful engagement. Connected with this point, it has often been debated whether it is not the “rest” *per se* but the “change” in the nature of a current activity that leads to a recovery of mental functions. Helton and Russell (2015) have examined this particular aspect. The individuals were administered either with a baseline condition (no rest), a pure rest condition, and several “change” conditions, including typical mental operations (e.g., letter-matching task, etc.). As a result, performance was best for rest, worst for the continuous condition, while the other (“change”) conditions were somewhere between the extremes, indicating that any change in a task cannot compete with having rest, which again means that rest is always the best option to recover (even though experimenters usually lack precise control over what exactly participants do when instructed to “simply rest”).

The strategic model bears an important implication that is pertinent to pause research. From any straightforward formulation of a non-strategic model, the observation of a time-on-task decrement would indicate a decline or total depletion of resources (Baumeister et al., 1998; Hagger et al., 2010; Inzlicht and Schmeichel, 2012). In contrast, the strategic-resource model incorporates that individuals take preventive measures to either economize resource expenditure, or doing this rather impulsively, for example, when they suddenly experience effort (Thayer et al., 1994; Inzlicht et al., 2018; Hoffmann et al., 2021). Contemporary theorists such as Thayer (1989), Matthews (2021) proposed that it is the internal experience of states of the two aspects “energy” and “tension” that serves as the “tachometer” indicating whether to save or to spend effort to the task at hand. Such an approach automatically implies the question of how these indicators can be measured, either as emotions (experienced effort) or behavior (experienced lapsing), or a combination based on somatosensory experience and self-observation, arising from a stumbling of performance fluency at some point during the task (Kerr, 1973; Dreisbach and Fischer, 2011; Langner et al., 2011; Steinborn et al., 2016). In his pioneering work, Smallwood et al. (2004) introduced a technique where individuals are occasionally asked during the session whether they were still focused on the task or whether their thoughts were wandering elsewhere (on-task vs. off-task). Across numerous tasks and contexts, it was reliably found that individuals were far from being perfect as indicated by the proportion of “off-task” moments, typically increasing from about 20% to around 50% during a testing period, irrespective of whether task performance showed a decrement or not (e.g., due to practice effects), which is one of the reasons why only a few studies were able to show a correspondence between self-reports and the time-on-task effect in performance.

### 4.3 Psychological Satiation/Reactive Inhibition

Although the theory of mental satiation is a highly relevant framework with historical roots in the field theory of Kurt Lewin (1890–1947), it is surprisingly underrepresented today. In this perspective, the critical variable that is relevant to performance is not a hypothetical volume of an energetic reservoir (reduced by work and replenished by rest), but the level of accumulated mental satiation that is experienced and considered the main driving force of behavior (e.g., Mojzisch and Schulz-Hardt, 2007; Kruglanski et al., 2012, 2014; Kurzban et al., 2013; Häusser and Mojzisch, 2017; Krishna and Strack, 2017). The satiation model therefore utilizes a diametrically opposing metaphor when compared to the resource model. This becomes clear when one compares the two following everyday phrases. (1) *The engine runs until the fuel supply is exhausted*. (2) *The engine runs until it has run hot* (i.e., exceeded a critical temperature). In the first example we have a *preconditioning parameter while* in the second one we have a *delimiting parameter*, but despite that, both metaphors may allow for similar predictions. Continuous activity leads to an increase in mental satiation, while breaks lead to a decrease in the same. This implies that one could derive exactly the same predictions based on a resource model or the satiation model, which is why these approaches should not, in a strict sense, be contrasted as opposing theories. Rather, they represent separate cultures that, despite having a common essence, differ partially in experimental approach as well as in somewhat different weighting of key arguments (Greenwald et al., 1986; Greenwald, 2012). Studies on psychological satiation, for example, typically put strong emphasis on the aspect of subjective experience as well as on those variables that contribute to or influence experience.

Psychological satiation is a phenomenon that arises when an action is carried out frequently and in a repetitive manner, so that the activity, which might initially be perceived as neutral, is increasingly perceived as being aversive in the course of continuous repetition. This occurs mainly in task types that consist of homogeneous and repetitive forms which are not intrinsically motivating by themselves (Pieters, 1985; Donk and Hagemeister, 1994; Van Breukelen et al., 1995; Neubauer and Knorr, 1998; Steinborn et al., 2018). Typical tasks that are very well suited to induce satiation experimentally can be found in the classic psychometric instruments for measuring active forms of sustained attention, the so-called speed tests, sometimes also termed concentration tests. Lewin (1928) assumed that the persistent and excessive repetition of the very same action over and over again undermines the built-in tendency of any cognitive system to strive for a gain of information and the experience of agentic competence, whereby a resistance gradient develops against the further continuation of the same action, which is subjectively experienced as aversive. However, when individuals are instructed to continue the task over a longer period of time, an increasing conflict develops between two opposing tendencies that can only be resolved by exerting willpower. Although individuals experience aversion as a cause, Lewin considered it a perceivable indicator in terms of a phenomenological by-product of the internally created tendency (Robinson and Clore, 2002; Strack and Deutsch, 2004). More recently, Mojzisch and Schulz-Hardt (2007) have studied the determinants of mental satiation in various contexts, based on field theory. In one series of experiments, groups of individuals were instructed to complete speeded test of different workload levels over a period of 20–40 min, using a loaded mental addition test (Düker, 1949), and the subjective experience was assessed before and after the test. It was found that a high level of satiation developed particularly when the task did not allow for any resting by mind wandering, that is, when it was both occupying and repetitive.

Based on classic theorizing on accumulating mental satiation and the resulting distraction as well as on the compensatory function of rests, Van Breukelen et al. (1995) presented a psychometric model to characterize and predict performance and performance fluctuations during sustained mental work. In essence, it is posited that the averaged reaction times (typically RT mean) are composed of two components: responses emitted in the state of mental *focus* and those emitted in the state of *distraction*. The model is a generalization of earlier psychometric models of cumulative inhibition, which are based on broadly similar assumptions but different in their specific statistical parameters. The theoretical framework of the inhibition model is Hull’s (1943) theory of reactive inhibition, and its implementation in the Rasch model for speed tests (Rasch, 1980; Pieters, 1985; Baghaei et al., 2019), which postulates that during ongoing monotonous processing, a kind of negative drive develops that forms an opposing gradient to the current goal orientation. In the context of performance testing, this can be seen as a “distraction tendency”. The gradient increases as a function of the throughput processed and immediately leads to distraction when a certain threshold is reached. The inhibition tendency thus increases monotonically during task processing and decreases during periods of distraction or during rest breaks.

Some studies are particularly suited to highlight theoretically fruitful research approaches in this context. For example, Sanders and Hoogenboom (1970) presented their participants with a six-choice RT task characterized by a rapid pace (response-stimulus interval = 60 ms) with either a continuous work or a rest-pause condition. The digits 1–6 served as targets and were mapped to six separate buttons. Responses became faster on average in the rest-break condition, while they remained the same in the continuous work condition. Furthermore, a cumulative distribution function (CDF) analysis revealed that the two conditions did not differ in terms of the fastest, but only in terms of the slowest CDF percentiles. Sanders and Hoogenboom (1970) argued that this reflects that rest breaks proactively prevent the occurrence of mental blocks and in this way reduce performance variability. This interpretation is consistent with earlier suggestions (Jersild, 1926, p. 34). More recently, Steinborn and Huestegge (2016) examined the effect of rest on performance and experience as a function of the factors “rest,” “demand,” and “time on task.” As a result, rest (vs. no rest) had beneficial effects on performance, which increased with time on task and was more pronounced for hard than for easy arithmetic. The CDF percentile analysis revealed that rest particularly reduced the frequency of dropouts and lapses. The pre–post assessment of experience revealed a differential pattern: while energy and engagement tended to decline, there was no effect of tension and distress. Taken together, these studies emphasize the importance of distributional analyses to draw relevant theoretical conclusions here.

### 4.4 Complicated Arguments in Current Theorizing

The majority of studies theorizes (in some or the other way) on grounds of resource models borrowed from the cognitive-experimental literature on multitasking research. An essential detail of particular models concerns the distinction of a variable vs. a fixed volume of capacity, with the former assuming a mobilization of capacity by immediate demand and the latter assuming an alternation or dynamic partial allocation of capacity between channels processing task-related vs. task-unrelated information. Another potentially important detail concerns the role of how feelings (before or during) task processing affect performance levels (e.g., energy, tension, motivation). The corresponding research traditions can be classified into three categories, (1) considering feelings as phenomenological by-products of cognition, (2) viewing them as a sort of “tachometer” of internal state indicating room for vigorous action or need for recuperation, or (3) as the underlying “cause” of the observed performance differences. Despite these pure categories, a transactional perspective would argue that while feelings may primarily have an indicator (tachometer) function, the mere act of reading out the internal state can by itself lead to distraction or conflict, similar to that in multitasking situations. Accordingly, an observed performance decline can actually be the result of distraction and self-referential processing (Wells and Matthews, 1996), or more precisely, from the act of monitoring and comparing actual values with an internal standard, and from subsequent evaluation or self-regulation (Carver and Scheier, 1990; Strack and Deutsch, 2004; Hewig et al., 2011; Krishna and Strack, 2017; Steinborn and Huestegge, 2020).

### 4.5 Mobilizing Capacity vs. Routing Channels

According to a mobilization model of sustained–attention performance, capacity is primarily demand-driven, which means that it is the immediate demands of the task that triggers capacity supply and not a deliberate decision. To a certain extent, it can be controlled at will, for example, when advance information is provided (Brown and Braver, 2005; Botvinick and Braver, 2015), or by a prompt (or reminder) to increase focus (Strayer and Kramer, 1994; Kleinsorge, 2001; Falkenstein et al., 2003; Steinborn et al., 2017), though it is impossible to control it precisely or to keep it steady. In a situation that emphasizes the aspect of “maintaining” performance levels over extended time periods, precise control would require a time-scheduled capacity threading between states of focusing and those of monitoring over the task period (cf. Craik, 1948; Humphreys and Revelle, 1984; Van Breukelen et al., 1995; Fernandez-Duque et al., 2000; Steinborn et al., 2017). This means that capacity varies over the duration of the task and thus can be characterized by a hypothetical ratio of utilized and spare capacity. According to Kahneman (1973), such variation is due to the fact that the allocation policy is not always set on focusing but sometimes sways capacity to other activities (monitoring or mind wandering), resulting in slower or even sluggish responses during these periods. Such a view of intermittent resource allocation to active operating vs. passive monitoring provides a natural way to explain the trial-to-trial response-speed variability that is commonly observed in reaction–time series (Flehmig et al., 2007a; Steinborn et al., 2016; Klein and Robinson, 2019).

A formal way to represent the aspect of performance fluctuation is to model data within the framework of the mixture-models type of speeded performance (Pieters, 1983, 1985; Miller, 2006; Schwarz and Miller, 2012). Basically, it is assumed that observed performance (i.e., reaction time) is composed of trials where the individual was under a state of focusing or a state of reduced focus (e.g., through non-registered rest, either enforced or taken). There are also many accounts that rather implicitly (and in a less formal way) refer to a similar idea without explicating the precise mechanisms of how exactly this is reflected in performance parameters (e.g., Humphreys and Revelle, 1984; Jensen, 1992; Leth-Steensen et al., 2000; Stuss et al., 2003; Robinson and Tamir, 2005; Flehmig et al., 2007a; Cheyne et al., 2009; Thomson et al., 2015). At a more sophisticated level, response variability is studied by analyzing the individual response-time distributions, often computed as a vincentized percentile function. Typically, reaction-time distributions tend to be leaning toward the right side (i.e., corresponding to the slower percentiles), in other words, to having a long tail toward the right. However, there is an important distinction that is often misunderstood in the literature. Precisely, to say that an experimental factor produced an effect on RT variability (i.e., to say it affects the ratio of focused vs. non-focused trials) requires that the factor has a selective effect on the slower percentiles of the distributive function that goes beyond mere scaling variability (De Jong et al., 1994; Miller, 2006; Steinborn et al., 2017), as would be indicated by the RT coefficient of variation (Flehmig et al., 2007a).

A capacity-mobilization model of sustained-attention performance can be characterized by some important key aspects: First, the hypothetical value of “capacity” is a latent (or theoretical) variable that is represented by the empirical indicator (or performance) variables, that is, in the speed of processing or the throughput of information processed per unit of time (Thorne, 2006; Szalma and Teo, 2012; Vandierendonck, 2017; Steinborn et al., 2018). Further, the mental resources devoted to continuous performance are essentially limited in two ways, (a) that the individuals punctually engage is only one mental activity, and (b) that they can maintain engagement for only a brief period. Task-relevant processing is referred to as utilized capacity and indicated by performance, while task-unrelated processing is referred to as spare capacity, which is the residual that can only be inferred indirectly (from the lack of optimal performance). The relation between both (i.e., the spare–utilized capacity ratio) varies across a sequence of trials, so that as individuals engage in task operations, spare capacity is reduced at the costs of utilized capacity, and vice versa, when they disengage from the task-relevant operation (i.e., microbreaks, either enforced or taken), utilized capacity is reduced at the cost of spare capacity (Steinborn and Huestegge, 2016, 2017, 2020). To know the dynamics of these alternation between spare and utilized capacity, it would be necessary to inspect not only the distributive function but also the time series of reaction times, separately for each individual. With this respect, only a very few studies examined this in greater detail (e.g., Kraepelin, 1902; Bills, 1931; Laming, 1979; Cheyne et al., 2011).

While a spare–utilized capacity model is both prudent and parsimonious in its base assumptions, more recent theoretical proposals seem to overstretch their structural basis, which generates a problem when it comes to using theory for deriving predictions. For example, Thomson et al. (2015) made an attempt to assume that an exactly equal amount of capacity alternates between two processes, one devoted to the task-related and the other reflecting task-unrelated processing (i.e., mindwandering), and over a series of trials, individuals are assumed to alternate between these two channels, while the capacity remains equal across all trials, thereby refuting any process of mobilization completely. According to this conception, performance is measured by standard parameters, while mindwandering occurs either by (passively) drifting off or by (actively) being on pause as assessed by self-report probes. The proportion of these conditions is assumed to change over time (i.e., the proportion of “off-task” conditions increases) which gives a measure of the amount of mindwandering, that is, not being on task. However, a problem here lies in a confusion of using natural language and theoretical formalism when speaking about performance effects. In natural language, one would agree that the brain does not consume more energy overall during phases of mental work as compared to alternating phases of mindwandering (but see Gailliot et al., 2007, for an opposing view). In the same vein, everyday language would link the term mobilization to changes in physiological activity, which is not addressed in the formulation of a spare–utilized capacity model. The problem is that any formal model that specifies capacity for alternative hypothetical processes (e.g., A = on-task; B = off-task) creates a problem in the formal structure in a way as the capacity budgeted for the on-task process is determined by the performance measures while those budgeted for the off-task process is indicated by the self-report probe trials. These self-reports give an indication that the individuals were not focused but absentminded, but they do not deliver an equivalent that allows to determine the capacity required during both alternating phases (Miller and Ulrich, 2013).

In summary, we argue that a formal model must be able to specify the degree of engagement of both the task-related and the task-unrelated process, and it must provide rules for determining the behavioral indications signaling this engagement. This is not possible in a model case where performance can be measured only for one task, while the other process (i.e., rest pauses, mindwandering, etc.) is indicated only indirectly, by the absence of a performance optimum (Van Breukelen et al., 1995; Miller and Ulrich, 2013). In order to be able to formulate a model that allows us to predict the direction or amount of capacity change during time on task, or afterward during rest breaks, it is crucial to link the theoretical variables to a connected measurement concept that specifies how the values of the model parameters are indicated by behavior and performance (most often performance speed and accuracy). Further, a measurement theory must also include a specification of performance characteristic trade-offs that presumably covary with the hypothetical state of mental fatigue (or overload, etc.). In most theoretical accounts, it is rather implicitly assumed (or simply presupposed) that both performance speed and accuracy deteriorate over time, indicating reduced mental efficiency, while pauses are assumed to restore mental efficiency. Empirically, it is often found that information processing slows down with no substantial effects on accuracy, and sometimes a speed–accuracy trade-off is observed (e.g., individuals become faster but more impulsive). Another aspect concerns the use of averaged performance parameters. Since mindwandering in continuous performance tasks is particularly evident in the slow percentiles of the reaction time distribution, it would be essential to additionally inspect time series data (Laming, 1979; Cheyne et al., 2009). In the field of rest-break research, these aspects are lacking, and in fact there are currently only a few studies that actually address these aspects in some or the other way (as outlined above).

## 5. Methodology, Design, and Psychometric Measurement

As aforementioned, there are numerous theoretical approaches, each of them highlighting different aspects of strain and recuperation, and each of them differing in the dynamics and time scale of these variables. In our assessment of the literature, there is a chaotic plentitude of theoretical proclamations available but hardly any testable model that would allow us to make straightforward predictions about performance effects. In view of this consideration, we worked toward evaluating the existing theoretical proposals with respect to their argument structure, completeness, and dialectical implicature. In our judgment, they can be characterized as historically grown research traditions, each with its own specific peculiarities, norms, and conventions, and each using reasonable conceptual metaphors at the underlying core (Ferguson and Heene, 2012; Greenwald, 2012; Proulx and Morey, 2021). Despite this, there are two practical questions for a researcher interested in studying mental fatigue and its restoration by rest breaks, that is, how to put all parts into relation, and, further, how to utilize the existing theoretical structure for deducing reasonable hypotheses. We argue that a proclaimed theoretical model is eligible only if it makes mechanistic (and falsifiable) predictions that are derived in a straightforward and non-ambivalent way, and further, a theoretical delineation must include a connected measurement model that specifies how the predicted effects of the experimental factors are reflected in performance measures. Finally, a theoretical proposal must waive any additional (specifying) assumptions if they on principle cannot be tested empirically, or are not accessible to measurement. In effect, a reduced and sharpened language when making predictions about performance effects would improve knowledge in the field by reducing redundancy and by creating a link between encapsulated subcultures, and would connect the knowledge and methodological repertoire between these separated fields of research (Ferguson and Heene, 2012). In the following, we will reflect on some of these issues in more detail.

### 5.1 The Experience–Performance Connection

As noted, all existing theoretical approaches can broadly be classified into two groups, which we term “*resource model*” and the “*satiation model.*” According to the resource model, mental work leads to a decrease in the energetic reservoir, while rest breaks lead to an increase. According to the satiation model, mental work leads to an accumulation of satiation, but rest breaks lead to a reduction. In their simplest form, both model categories utilize a linear metaphor that is merely inversely poled (either as a permitting or delimiting condition). One particular difference lies in the emphasis on aspects of subjective experience. While the satiation model ties the loss of perceived intrinsic motivation to objectively measurable performance, the resource-model theorizes on the reduction of an energetic reservoir by mental work, and any reference to subjective experience is neither necessary nor mandatory. In fact, the proposal that experience and performance is connected or even causes each other is by no means trivial, at least it cannot automatically be taken as a presumption (Langner et al., 2010; Matthews, 2021). The literature is completely mixed in this regard. While a substantial number of studies reported a correspondence of pre-tested subjective state (e.g., energy, motivation, etc.) and subsequent performance, only a few were able to demonstrate a correspondence of the time course of these variables. For example, Thomson et al. (2014) showed a congruent temporal trajectory of self-reported mind-wandering tendencies and performance in one (relatively easy) task, while there was an opposing tendency of these measures in another (more complex) task. Clearly, the arrangement in one task was simpler and more repetitive than in the other one, providing fewer possibilities for procedural learning (e.g., Compton and Logan, 1991, see **Figure 2**; Healy et al., 2006).

### 5.2 Primary Measurement Artifacts

There are many aspects to be taken into consideration when conducting an experimental study on the effect of rest breaks on feelings and performance. Most of them relate to design, tasks, and performance measurement and are part of the basic knowledge repertoire of a skilled experimenter (Greenwald, 2012; Miller and Ulrich, 2013). But there is one aspect that pervades the entire research, the potentially concealing effects of test practice and item-specific learning (Flehmig et al., 2007b,^2010^), which is difficult to control properly (Donk and Hagemeister, 1994; Hagemeister, 1994, 2007). Precisely, performance-based tests such as typing, cancelation, or mental-arithmetic are typically used as propagation tasks to induce ego depletion (Hagger et al., 2010; Vohs et al., 2021). These test forms are typically administered as self-paced versions thus requiring sustained-information transfer (Humphreys and Revelle, 1984; Steinborn et al., 2018), they are highly demanding, and most crucially, they achieve exceptional test–retest reliability, since many items can be processed per unit of time. Yet, it should be considered that the time-on-task effects on performance do not always produce a global response slowing, and further they are (to an unknown degree) subject to procedural learning. Finally, fatigue might affect these processes differently at different points in practice (Healy et al., 2004, 2006). Some authors opted for the use of simple tasks like the sustained-attention-to-response test (SART, Manly et al., 1999; Seli et al., 2013; Head and Helton, 2014), the psychomotor vigilance test (PVT) (PVT, Dinges et al., 1997; Steinborn et al., 2016; Unsworth and Robison, 2019), or even detection-based vigilance tests as measure of choice (Warm et al., 1974; Thomson et al., 2016). While these tasks are less susceptible to learning, due to their simplicity, the items in these tests are typically spaced in time so that a relatively low number of trials is obtained during testing as compared to self-paced tests, where each item follows after the previous one with no time in-between trials. Consequently, test–retest reliability has often been found to be a weakness of the former (vigilance tests) as compared to the latter (self-paced tests) if one considers the economy of testing (Miller and Ulrich, 2013; Steinborn et al., 2018).

### 5.3 Design Methodology

Many published studies actually suffer not merely from methodological weaknesses of aspects connected to measurement methodology, but also from inadequate (or incomplete) design issues. As noted, fundamental to the study of rest breaks is that several distinct effects of rest can be distinguished with a slightly different meaning of each of them, not to speak of the contextual effects that often change the dynamics of these effects, for instance, when testing effects of rest under sleep deprivation (Wilkinson, 1959; Sagaspe et al., 2006; Bratzke et al., 2009, 2012). This is not considered in most of the literature. Essentially, rest-break effects as obtained via an experimental design that contains a time-on-task condition can be divided into global and local difference effects (cf. Steinborn and Huestegge, 2016). In order to examine the global effect of rest on performance, the performance trajectory between the control (no-rest) condition and the critical (rest) condition is typically examined. This can be done by using a group-based design where the critical conditions are compared in terms of a between-subject comparison. The global benefit of rest is obtained as the relative difference in the time course of the performance curve (i.e., the slope of the time-on-task effect), as indicated by the relevant performance parameters. The local-rest effect can (for obvious reasons) only be studied in the critical group (where rest-breaks are included). Here, the performance immediately before and after rest (i.e., the pre–post effect) of only the critical group is contrasted. This approach is formally equivalent to an analysis of trial sequences surrounding a critical event, such as errors (Brewer and Smith, 1984; Steinborn et al., 2012; Jonker et al., 2013), or attentional lapses (Bills, 1931; Bertelson and Joffe, 1963; Steinborn et al., 2016).

### 5.4 Psychometric Performance Measurement

While the effects of rest can basically be studied in any task form, speeded tests (e.g., mental arithmetic, letter cancelation, coding, etc.) are particularly suitable for performance measurement (cf. Van Breukelen et al., 1995). Since they require continuous work and because items are not spaced but rather compressed in time (items follow immediately after each other), they usually provide superior test reliability (Steinborn et al., 2018; Wühr and Ansorge, 2019). Individuals in these tasks are typically required to continuously respond to a series of successively presented targets, with each target following immediately after responding to the previous one, with no feedback given after errors. It should only be noted that individuals can efficiently detect any errors that they make, and they would even do so even when instructed to ignore them (Maylor and Rabbitt, 1995; Steinborn et al., 2012). One advantage is that they can be administered both as computerized versions of paper-and-pencil forms without changing the nature of the task (Van Breukelen et al., 1995; Steinborn et al., 2018). Since these tests offer the opportunity to simultaneously consider several performance aspects, they deliver some additional information about the working style. Typical measures are the speed, accuracy, and variability (Pieters, 1983, 1985; Flehmig et al., 2007a), or combined-efficiency indices (Thorne, 2006; Szalma and Teo, 2012). To summarize, a task must be applicable to experimentally study rest breaks, which means in the first place that it must put some strain on the individual, and it must allow for a reliable performance measurement. Although this may seem trivial, this condition is not met in the majority of empirical studies. However, without precise measurement, a sufficient number of trials, and adequate performance output variables, it will not be possible to derive correct theoretical conclusions from empirical results (Miller and Ulrich, 2013; Steinborn et al., 2018), and a proliferation of false conclusions would finally produce a chaotic plentitude of contradictory knowledge clusters rather than a systematic understanding of the underlying mechanisms of rest.

## 6. Determiners of Rest-Break Effects

Although it seems natural to review the empirical evidence in a certain field in the light of the critical factors determining the effect size including moderator variables, we found it impossible to do so in the field of rest-break research, for several reasons. First, the term rest is a bit of an umbrella term that has a bearing on several aspects differing in time and context (cf. Antonovsky, 1979; Kaplan and Kaplan, 1989). Further, and despite an evidently vast body of literature, the methodical approach is at the same time completely underdeveloped with respect to tasks and measures (Steinborn and Huestegge, 2016). That is, the majority of studies are extremely heterogeneous with regard to context, design and implementation of critical means and measures, and often suffer from the constraints and practicalities imposed by the adopted field approach (Meehl, 1967; Wendsche et al., 2016; Wendsche and Lohmann-Haislah, 2017; Scholz et al., 2019). Moreover, there are substantial methodological weaknesses and inadequacies of many reported studies in this domain, which relate to aspects such as the employment of arbitrary tasks and/or unreliable or unaudited performance measures, or simply an insufficient number of trials (Miller and Ulrich, 2013; Steinborn et al., 2018). Despite this, there are some crucial principles which are essential to consider when adopting the experimental approach to study rest breaks.

First, the response–stimulus interval is of importance, and effects of rest breaks are likely more pronounced when this interval is short than when it is long, or when a rhythmic pace is administered between them (Wilkinson, 1959, 1990; Sanabria et al., 2011; Steinborn and Langner, 2012). Second, if a study seeks to induce a depletion of resources by a task, it must critically be ensured that the individuals are working at full tilt when performing this task (Kleinsorge, 2001; Miller and Ulrich, 2013; Steinborn et al., 2017). In other words, the time trajectory of performance can only meaningfully be interpreted when it can be ensured that the individuals are not adopting preventive strategies of withholding performance. While this can more easily be achieved in laboratory studies, and can even be boosted by the presence of an experimenter or by reminding instructions to do best, this condition is typically lacking in many field studies (Scholz et al., 2019). A recent study of Johnson et al. (2019) that contains several flaws with respect to design and measurement might serve as an example to demonstrate this problem: In this study, one group of individuals was administered with a walking condition in a nature environment while the other was in an urban environment, with performance being tested afterward for both groups. The first problem here is that there is no pre-to-post measurement. This is suboptimal at the level of measurement and design, but can probably be compensated partly if the sample is large and comparable between groups. The second point concerns the use of a 30 min battery of several tests administered consecutively (i.e., after each other). This, however, is a severe flaw in the design rendering clear-cut interpretations difficult. *Why is this so?* Because each task of a battery of consecutive tests is affected by the preceding task which propagates depletion or fatigue (in unknown proportion) on the subsequent one, so that a test later in the sequence of the test battery is likely not affected by the walk (in nature vs. urban environment) but by the immediately preceding task of the test battery. Together, these issues were discussed only to exemplarily highlight the relevance of paying attention to a number of issues affecting research on rest break effects.

There are a number of fundamental questions that immediately come to mind when theorizing on the effect of rest on mental performance, which basically address the three key aspects length, distribution, and kind of content or activity, respectively. There are reoccurring themes that were already asked by even the earliest authors in the field (Manzer, 1927; Lewin, 1928; Wilkinson, 1959; Eysenck, 1969; Rohmert, 1973a), though it is difficult to give a universal answer to it: How long should a rest be to be effective? How should rest periods be spent? How restful is a change of task? On the basis of the literature, it is possible to summarize some general rules applicable to rest periods. For ordinary periods of 30–120 min of mental work using either speeded test paradigms or vigilance tasks, rest periods are optimal in the range of 3–10 min. It is certainly possible to reveal differences even within this range, but this may be dependent on specific design characteristics (Helton and Russell, 2012; Lim and Kwok, 2016; Lim et al., 2016). Longer periods are detrimental because the individual is at risk of losing the general mindset to perform at maximum level, and accordingly, will leave the individual in a rather unprepared (i.e., *restart cost effect*) state to resume the task optimally. Note again that the terminology in the restart-cost literature differs between experimental disciplines, or has a more specific meaning in other (sub)disciplines such as multitasking research (Wylie and Allport, 2000; Altmann, 2002; Janczyk et al., 2008; Kiesel et al., 2010; Vandierendonck et al., 2010).

Motivation or incentives are especially effective only if the individual is reminded of them during the task period simply because individuals tend to quickly forget initial task instructions over long testing periods (Altmann, 2002; Steinborn et al., 2017; Massar et al., 2018), or when the task itself has a game-like structure (Los et al., 2013), or has the “completable” property (Zeigarnik, 1927). A stronger focus and maintenance of high performance levels seem to tap more strongly into mental resources, thus yielding a stronger decline of performance with time. This, on the other hand, directly implies that lack of focus (or unwillingness to do best) can under some circumstances result in reduced or even absent time-on-task effects on performance, a potential problem that is especially important to keep in mind in the study of individual differences. For example, Lim et al. (2012) analyzed individual differences in the performance decline during the psychomotor vigilance task (PVT), with a focus on genetic polymorphisms related to attention deficit disorder (ADHD). Although the authors originally expected a more severe decline of performance in the vulnerable (vs. their counterparts) group, the results were in the opposite direction, simply because the individuals who were slow right from the start did not show the expected decline in performance over the testing period.

Most critically, monotonous work and work that is highly continuous requires the most frequent rest (Van Breukelen et al., 1995; Mojzisch and Schulz-Hardt, 2007; Häusser and Mojzisch, 2017). However, also in this context it might be stated that frequent (but relative brief) rest is more critical than longer rest. Further, the length and frequency of implanted rest breaks is of importance, too, and exactly this aspect was already considered by earlier studies. For example, Bills (1943, pp. 113–129) evaluated the body of empirical evidence of experimental studies at his time concluding that rest breaks should be brief (3–6 min) but frequent, and should not exceed 8 min in length. Bills argued that any increase beyond the optimal length could result in a decrease in basal activation level, or task-related activation level, respectively (Van Breukelen et al., 1995; Steinborn and Huestegge, 2016). Regarding the position in time during the testing period, the rest is more effective when given late during a test, relative to when given early, though there are only a few studies that examined this aspect via proper design (Ralph et al., 2016; Steinborn and Huestegge, 2016). How the rest period should be spent is certainly relevant, too. The satiation model would suggest that this primarily depends on whether the change task provides distance from the basic task, or whether some property of the situation (i.e., walking) naturally changes the attention policy (Cao and Händel, 2019). Yet, there are no metrics available to objectively determine the degree of similarity of tasks (Norman and Bobrow, 1975; Navon and Gopher, 1979; Meyer and Kieras, 1997a,b). Accordingly, the empirical evidence is rather unclear. Yet, a mere change in the task at hand is unlikely to be more effective than rest, since recuperation is most likely when the shift occurs from a performance-based activity to a period that provides sensations that can be processed with effortless attention that is not tied to goals (cf. Humphreys and Revelle, 1984; Kaplan, 1995; Greenwald and Gillmore, 1997; Mojzisch and Schulz-Hardt, 2007; Salvucci and Taatgen, 2008; Colzato et al., 2012; Kurzban et al., 2013; for theoretical viewpoints). As mentioned earlier, Helton and Russell (2012, 2015) pointed toward the difficulty to control for (micro-) rest periods between subsequent tasks, typically not registered as such, arguing that maximally “pure” rest is, in most cases, likely the best option as compared to a change in the task whatever the task is (e.g., Ariga and Lieras, 2011; Lim and Kwok, 2016; Steinborn and Huestegge, 2016).

## 7. Conclusion and Future Directions

In the present paper, we aimed to present a structured overview of both theory and empirical findings related to effects of rest breaks. On the backdrop of this summary, we developed a set of recommendations that should be able to provide some guidance for future studies in the field. We are currently living in a world characterized by acceleration on many fronts, which yields ever-growing task and performance demands, often requiring the execution of multiple tasks at around the same time (Engelmann et al., 2011; Wörle et al., 2021). In this context, it appears essential for corresponding research fields to focus on ways which render life in a multitasking world more bearable (Colzato et al., 2021; Hoffmann et al., 2021; Hommel and Beste, 2021; Kärtner et al., 2021), and the study of rest as a means to foster both performance and well-being represents a core endeavor that should clearly be explored further, but ideally on a maximally advanced level with respect to both theory building and methodology. The present paper was written with this objective in mind. From an applied point of view, many responses to important questions are still in their infancy. For example, typical rest breaks in working environments have fundamentally changed in nature, as nowadays most people devote their spare time whenever possible to the use of social media and web content using their smartphones or computers, and media coverage of such phenomena often emphasizes potentially serious distraction effects accompanying such behavior (Charlton, 2009; Ralph et al., 2014; Scheiter et al., 2014; Steinborn and Huestegge, 2017). Again, such discussions call for a more rigorous analysis of differential effects of type of rest. Finally, we predict that more research will be devoted to study interindividual differences, for example, regarding the efficiency of certain types of rest or regarding individual differences in spending spare time in the first place.

## Data Availability Statement

The original contributions presented in the study are included in the article, further inquiries can be directed to the corresponding author/s.

## Author Contributions

JK, LC, BF, and LH contributed to the supervision, discourse, debate, and theorizing. FS and MS contributed to the concept and writing the manuscript. All authors contributed to the article and approved the submitted version.

## Conflict of Interest

The authors declare that the research was conducted in the absence of any commercial or financial relationships that could be construed as a potential conflict of interest.

## Publisher’s Note

All claims expressed in this article are solely those of the authors and do not necessarily represent those of their affiliated organizations, or those of the publisher, the editors and the reviewers. Any product that may be evaluated in this article, or claim that may be made by its manufacturer, is not guaranteed or endorsed by the publisher.
